# Algal Bioactive Metabolites with Important Roles in Wound Healing

**DOI:** 10.3390/pharmaceutics18091104

**Published:** 2026-09-02

**Authors:** Tünay Karan, Çağrı Çağlar Sinmez, Sevgi Durna Daştan, Murat Çakir, Mücahit Seçme, René van den Hoven

**Affiliations:** 1 Department of Genetics, Faculty of Veterinary Medicine, Yozgat Bozok University, Yozgat 66100, Türkiye; 2 Department of History of Veterinary Medicine and Deontology, Faculty of Veterinary Medicine, Erciyes University, Kayseri 38039, Türkiye; cagrisinmez@erciyes.edu.tr; 3 Department of Biology, Faculty of Science, Sivas Cumhuriyet University, Sivas 58140, Türkiye; 4 Department of Physiology, Faculty of Medicine, Yozgat Bozok University, Yozgat 66100, Türkiye; 5 Department of Medical Biology, School of Medicine, Ordu University, Ordu 52200, Türkiye; 6 Clinical Unit of Equine Internal Medicine, Clinical Centre for Equine Health and Research, University of Veterinary Medicine Vienna, 1210 Vienna, Austria; prof.renevandenhoven@gmail.com

**Keywords:** algae, wound healing, bioactive metabolites, phytochemicals, good health and well-being

## Abstract

A wound is defined as disruption or destruction of tissue integrity. In order to support healing in wound management, a good wound bed free of necrotic tissue and infection is desired, but intensive chemical antiseptics will cause cell destruction and delay healing. In order for wound healing to be rapid, the nature and contamination of the wound should be taken into consideration and appropriate methods should be utilized. Today, many types of algae are frequently preferred as an alternative to medicine and are the subject of research. Since the metabolites contained in algae display several notable biological activities such as antimicrobial, anti-inflammatory, and antioxidant, they are a good option in wound treatment. Algae contain pigments, peptides, fatty acids, and polysaccharides that are crucial for wound healing. These compounds play vital roles at all stages of the healing process by accelerating cell proliferation, promoting collagen deposition, scavenging reactive oxygen species (ROS), and regulating key inflammatory cytokines. Furthermore, their unique physical and functional properties enable the development of novel bio-inspired wound dressings, hydrogels, and drug-delivery scaffolds. This review discusses the bioactive metabolites found in algae that are effective in wound healing.

## 1. Introduction

Algae are primary producers that can be found everywhere on earth but their main distribution areas are aquatic. They have sexual, asexual and most commonly vegetative reproduction systems. In the simplest shaped species, vegetative cells are similar to each other. Each cell can reproduce individually by dividing into two. Spores and gametes are formed in those that do not divide vegetatively. Since root, stem and leaf differentiation is not seen in algae, the thallus structure is mentioned. If cell divisions occur in a single cell row in the same direction, filamentous thallus occurs and this type is called trichoid [1]. When algae groups are compared with each other, it is seen that there are differences between them in terms of morphology, cytology, biochemistry and reproduction. Algae are classified according to their cellular structure and pigmentation. Although traditional classifications grouped blue–green algae (Cyanophyta), brown algae (Phaeophyta), red algae (Rhodophyta), green algae (Chlorophyta), diatoms (Chrysophyta), and flagellate algae (Flagellata) together under the general category of ‘algae’, a fundamental taxonomic distinction exists from an evolutionary biology perspective. While Cyanobacteria possess a prokaryotic cellular organization, all other algae groups are eukaryotic [2].

Algae, which play a key role in the food chain, are used in food, cosmetics, agriculture, medicine, pharmacy and various industrial areas. According to research, agar, alginic acid, organic acids and cellulose are obtained from algae. Alginates are the main ingredients of dressings, bandages and plasters. They are used in making pastries, marmalades, jams and ice creams, in preparing sausage casings and in preserving oily fish such as mackerel [3,4]. Polyunsaturated fatty acids such as omega 3 and omega 6 are noteworthy in microalgae. Some algae can be used for weight loss due to their protein, low-fat and digestible carbohydrate content. There are algae that contain special fatty acids such as γ-linoleic acid (GLA), eicosapentaenoic acid (EPA), docosahexaenoic acid (DHA), and arachidonic acid (ARA) [5]. *Spirulina* sp. is a cyanobacteria with a high protein content, used as a dietary supplement and also in the aquarium and poultry industry [6]. Algae are also beneficial as animal feed and fertilizer. They are used in cleaning domestic and industrial waste, and as a finishing agent and emulsion in the paint, paper, textile, plastic and leather industry. There is evidence that it can be widely used in goiter treatment and kidney diseases. It is used in reducing high fever, regulating blood circulation and balancing the immune system. Studies have shown that algae exhibit antibacterial, antioxidant, antiviral, anticancer and anthelmintic activities [7]. Astaxanthin and carotenoids such as β-carotene and lutein are strong antioxidants and algae containing these carotenoids are quite significant in medicine and food. The development of new pharmaceutical agents for wound healing, especially from algae containing biologically active secondary metabolites, is important in the pharmaceutical industry [8].

Wound is the loss of all or part of physiological activities in living tissue and the disruption of tissue integrity. Wounds can affect epithelial tissue as well as muscles, tendons, nerves, veins and even bone tissue in some cases [9]. Wounds can occur due to chemical and physical burns, trauma, metabolic disorders, nutritional disorders, drug interactions or animal bites. Wounds are classified into two different categories as acute or chronic. Acute wounds occur as a result of tissue loss following trauma or surgical procedures, and these wounds can be repaired over time [10]. Chronic wounds are wounds that progress irregularly due to disruptions in the anatomical and functional healing process and are not repaired in a timely manner. These wounds can be caused by a variety of factors such as metabolic diseases, immune system dysfunction, arterial and venous insufficiency, and burns. The healing procedure of chronic wounds is long and in some cases, it is seen that the wound does not heal completely.

Algae have anti-inflammatory, antioxidant, and antibacterial properties as a result of the bioactive metabolites they contain. It has been stated that extracts obtained from algae reduce inflammation and pain, prevent infections and facilitate wound healing. They also increase the formation of the extracellular matrix by promoting cell division, which is important for wound healing. It has been stated in the literature that some algae improve tissue remodeling, reduce scarring with high humidity and biocompatibility, and provide faster healing. The ability of algae to provide new formulations such as hydrogels, dressings and topical applications marks a new area of development in wound care technology. Based on these properties, algal metabolites need to be extensively investigated and evaluated for their potential use as drug carrier scaffolds or wound dressings. Pigments, peptides, fatty acids, and polysaccharides contained in algae are major contributors to both skin aging and wound healing [11,12]. In this review, the chemical structures, bioactive properties and roles of important algal metabolites in wound healing, care and dressing are discussed.

## 2. Wound Healing Mechanism

Wound healing is a dynamical process that begins immediately after injury, and cytokines and growth factors are effective in wound healing. Cytokines are protein molecules that are effective in the development and maturation of cells by providing communication between cells. Growth factors are a subgroup of cytokines. All wounds have basic wound healing stages, including hemostasis, inflammation, proliferation–epithelialization, and remodeling [13]. In the hemostasis stage, cytokines that provide information transfer between cells are released as a result of existing tissue damage and many complex events begin. When the vascular wall is damaged, platelets become active with the activation of Thromboxane A2 and active thrombin converts fibrinogen into fibrin. Thus, clot formation and bleeding decreases [14]. In the inflammation phase, neutrophils and other inflammatory cells reach the wound area and prevent infection. Macrophages kill and phagocytose bacteria. After the dead tissue and monocytes are cleaned, angiogenic growth factors are secreted and new vessels are formed, oxygenation of the area occurs and nutrition improves [15]. Fibroblasts are dominant in proliferation and epithelialization processes and produce collagen fibers, leading to increases in tensile strength and contraction. In this stage of epithelialization and contraction, the wound size decreases and the tissue becomes stronger. Remodeling is the last and longest phase in wound healing. In this phase, where collagen fibers remodel, type III collagen turns into type I collagen. Over time, the tension strength in the tissue increases to the maximum level and scar tissue formation is observed [14].

Wound healing is examined in three chief groups as primary, secondary and tertiary. In primary healing, complications are at a minimum level and wound closure is very fast. Scar tissue develops in a very small amounts. In secondary healing, healing occurs spontaneously without any procedure for the formation of granulation tissue. This can be applied to wounds requiring physical therapy, after surgical procedures and animal bites. Finally, tertiary healing is defined as delayed primary closure and is the closure of the wound lips after the wound is made suitable for primary repair and granulation tissue is formed [16,17].

Due to their unique bioactive constituents, algae-derived biocompounds have recently gained prominence in tissue engineering and wound care applications for their role in the wound healing process. Algae represent an exceptionally rich source of sulfated polysaccharides (fucoidan, ulvan, carrageenan), neutral polymers (alginate, laminarin), natural pigments (astaxanthin, phycocyanin, chlorophyll), and functional lipids. These bioactive metabolites possess the capacity to modulate cellular signaling and inflammatory responses at every stage of wound healing, from hemostasis to tissue remodeling. In particular, their ability to suppress inflammation by accelerating the M1/M2 macrophage phenotype transition, stimulate angiogenesis and fibroblast migration via VEGF and TGF-β signaling pathways, and limit tissue damage through antioxidant activity positions algae-derived matrices and hydrogels as ideal biomaterials [18,19,20].

## 3. Algal Metabolites and Bioactivities

Secondary metabolites are natural organic products that are not directly related to growth and development but are as important as primary metabolites. They are natural products that have therapeutic effects against human and animal diseases. In addition, it is understood that these compounds are generated when needed by chemical mechanisms, as they may have functions such as survival, defense, protection, and adaptation to the environment in living things. Secondary metabolites, which are important in various industrial areas such as plant resistance, food, health, and medicine, are divided into three groups according to their biosynthetic origins: alkaloids, terpenes, and phenolic compounds [21].

The most studied secondary metabolites today are terpenoids, alkaloids and halogenated compounds. Compounds obtained from red algae are generally halogenated. The presence of phenolics such as phenols, flavonoids and tannins in algae indicates antioxidant activity and free radical scavenging effect. Cyanobacteria (blue–green algae) produce nitrogenous compounds and cyclic polyethers with strong biological activity. Cytotoxic compounds such as dolastatin 10, aprotoxin A, and largazol are produced by cyanobacteria [22]. Curacin-A, with anticancer properties, was isolated from *Lyngbya majuscula* [23]. Symplostatin-1, with cytotoxic properties, isolated from *Symploca hydnoides* was reported to affect murine colon 38 and murine mammary 16c cell lines by inhibition of endothelial cell proliferation [24]. Dolastatin peptides, which are neoplastic agents isolated from *Caldora penicillata* and trigger apoptosis in lymphoma cell lines, were obtained from blue–green algae [25]. Goniodomin A, isolated from the dinoflagellates *Goniodoma* sp., is a powerful antifungal agent [26]. Capisterone A and B are triterpenesulfate esters isolated from the green alga *Penicillus capitatus* and are potent antifungals [27]. Peyssonol A and B are sesquiterpene hydroquinones with anti-HIV activity isolated from *Peyssonnelia* sp. [28]. β-bisabolene isolated from *Laurencia scoparia* is an anthelmintic halogenated sesquiterpene [29]. Chondriamide A, which is cytotoxic against colorectal cancer cell lines, was isolated from red alga *Chondria atropurpurea*. Recently, the number of compounds isolated from algae has increased and the identified compounds are given in Table 1, which have anticancer, antioxidant, antibacterial, and antiviral activities [30].

## 4. Algal Bioactive Metabolites Used in Wound Healing

### 4.1. Fatty Acids

Fatty acids found in algae act as components of membrane phospholipids and play a crucial role in wound healing by initiating an inflammatory response. One study reported that polyunsaturated fatty acids in the green alga *Parachlorella kessleri* have high antioxidant effects and that the results in wound and burn healing are remarkable. Omega 3 and omega 6 are fatty acids that cannot be synthesized in the body and must be taken from outside, and they are therapeutic in wound healing by stimulating collagen synthesis [61]. EPA and DHA polyunsaturated fatty acids have anti-inflammatory and antioxidant properties and prevent skin water loss [62]. Linoleic acid has been detected in *Chlorella vulgaris*, and gamma linolenic acid, linoleic acid, and palmitic acid have been detected in *Spirulina platensis* and *Spirulina maxima*, and it has been reported that these fatty acids improve the skin through softening [63,64].

### 4.2. Bioactive Peptides

Peptides are molecules with amino acid sequences that are often found in anti-wrinkle creams and provide a better appearance to the skin. Due to their diversity in formation and function, they are widely used both naturally and synthetically in biology and medicine. Cyclic peptides, which can be formed by amide bonds in a circular array between proteinogenic or non-proteinogenic amino acids, are of interest in wound healing therapy. In addition to standard bioactive peptides derived from various animal sources or synthetic routes, algal-derived peptides have recently gained significant attention as novel therapeutic agents for tissue repair. Algae (such as *Spirulina* sp., *Chlorella* sp., and *Porphyra* sp.) produce unique bioactive peptides and protein hydrolysates with strong antioxidant, anti-inflammatory, and immunomodulatory properties. For instance, specific peptides derived from red algae (*Porphyra yezoensis*) and phycobiliprotein-derived enzymatic hydrolysates from cyanobacteria (*Spirulina platensis*) have been shown to accelerate wound closure by stimulating fibroblast proliferation, enhancing keratinocyte migration, and scavenging reactive oxygen species (ROS) at the wound site. These marine algae-derived peptides offer eco-friendly, highly biocompatible, and non-toxic alternatives for soft tissue regeneration and modern wound dressings [65,66,67].

The antioxidant activity of algae-derived peptides may be related to peptide size. Peptides with hydrophobic amino acids such as Phe, Trp, Tyr, Ala, Val, and Leu scavenge free radicals. Acidic or basic amino acids such as Lys, Asp, and Glu can chelate metal ions. Aromatic amino acids such as Phe, Trp, and Tyr quench free radicals by direct electron transfer [68]. Some algal peptides with antioxidant activity are listed in Table 2.

**Table 2:** Antioxidant algae peptides in wound healing.

Algae	Peptide Sequence	References
*Chlorella vulgaris*	Val-Glu-Cys-Tyr-Gly-Pro-Asn-Arg-Pro-Glu-Phe	[69]
*Navicula incerta*	Pro-Gly-Trp-Asn-Gln-Trp-Phe-Leu-Val-Glu-Val-Leu-Pro-Pro-Ala-Glu-Leu	[70]
*Chlorella ellipsiodea*	Leu-Asn-Gly-Asp-Val-Trp	[71]
*Porphyra columbina*	Ala-Asp-Glu-Leu-Pro-Thr	[72]

### 4.3. Algal Pigments

**Figure 1:**
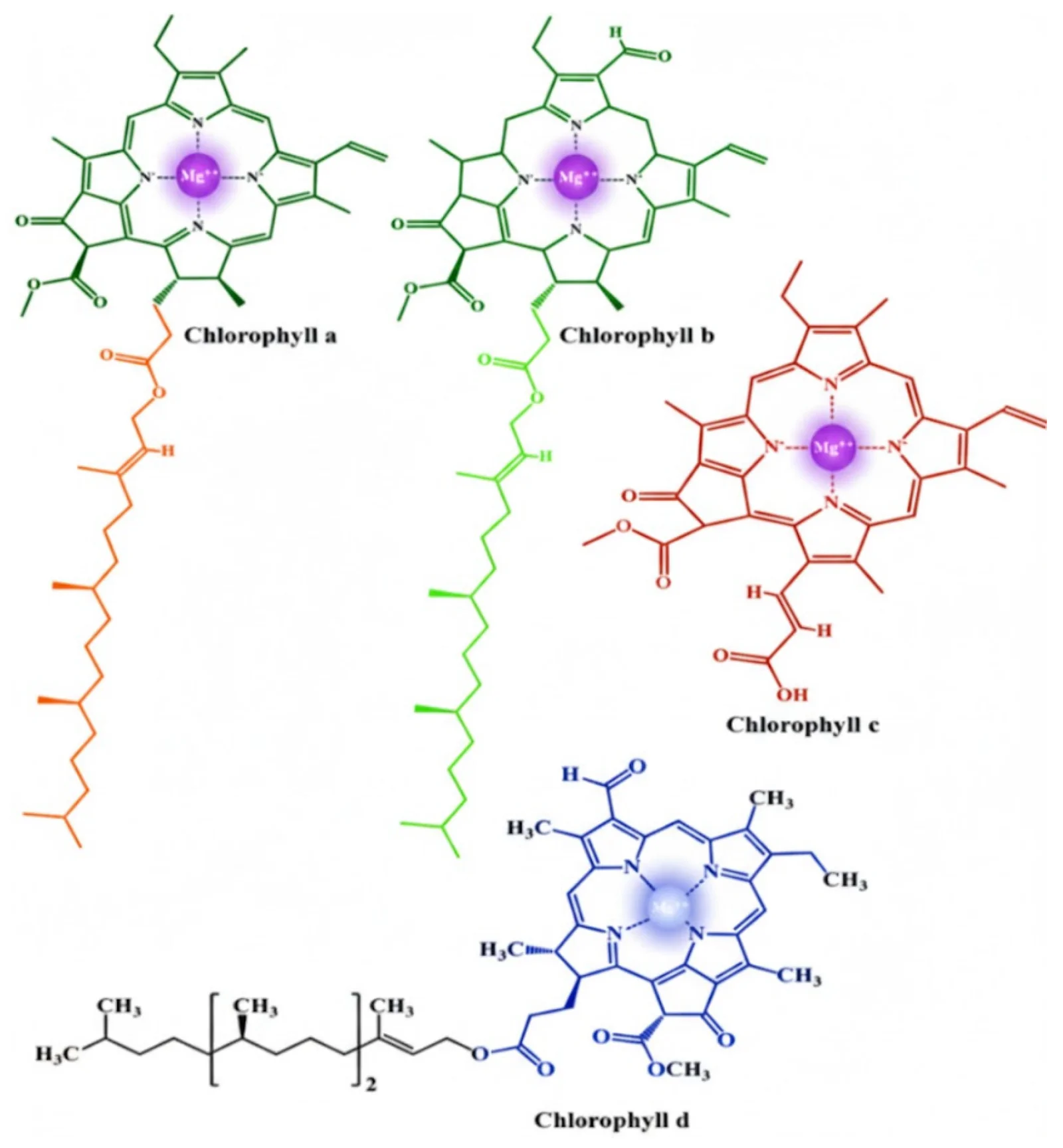
Chemical structure of chlorophylls.

Algae are potential sources of natural pigments, including chlorophylls, carotenoids and phycobiliproteins, and these pigments have been used in the food, cosmetic and pharmaceutical industries for many years [73]. The effects of various pigments obtained from algae on wound healing are given in Table 3. Chlorophyll, the photosynthetic greenish pigment found in algae, cyanobacteria and plants, contains a porphyrin ring and belongs to the main class of tetrapyrroles [74]. There are four main types of chlorophyll including chlorophyll a, chlorophyll b, chlorophyll c and chlorophyll d (Figure 1). Chlorophyll a is a blue–green pigment with maximum absorption between 660 and 665 nm required for photosynthesis and is the critical tetrapyrrolic pigment found in seaweed and cyanobacteria. Chlorophyll b is an accessory pigment and is a green–yellow pigment with maximum absorption between 642 and 652 nm. Chlorophyll c is a blue–greenish color pigment with maximum absorption between 447 and 452 nm. Chlorophyll d absorbs far-red light at a wavelength of 710 nm and is found in red algae and marine cyanobacteria [75,76,77].

Chlorophylls and their water-soluble derivatives possess unique biochemical activities that contribute significantly to tissue repair. Although traditionally known for their spectral absorption properties, current biomedical research reveals that they play a direct role in wound care through antimicrobial photodynamic therapy (aPDT), the regulation of ROS, and the stimulation of tissue regeneration. Upon light activation at specific wavelengths, chlorophyll molecules act as photosensitizers, transferring energy to molecular oxygen to produce singlet oxygen [78]. This localized oxidative burst induces rapid bacterial cell membrane damage and protein denaturation, effectively eradicating drug-resistant pathogens. Allophycocyanin and chlorophyll derivatives enhance the proliferation and migration of dermal fibroblasts, up-regulate collagen type I synthesis, and accelerate re-epithelialization by modulating transforming growth factor-beta (TGF-β) signaling [79].

**Figure 2:**
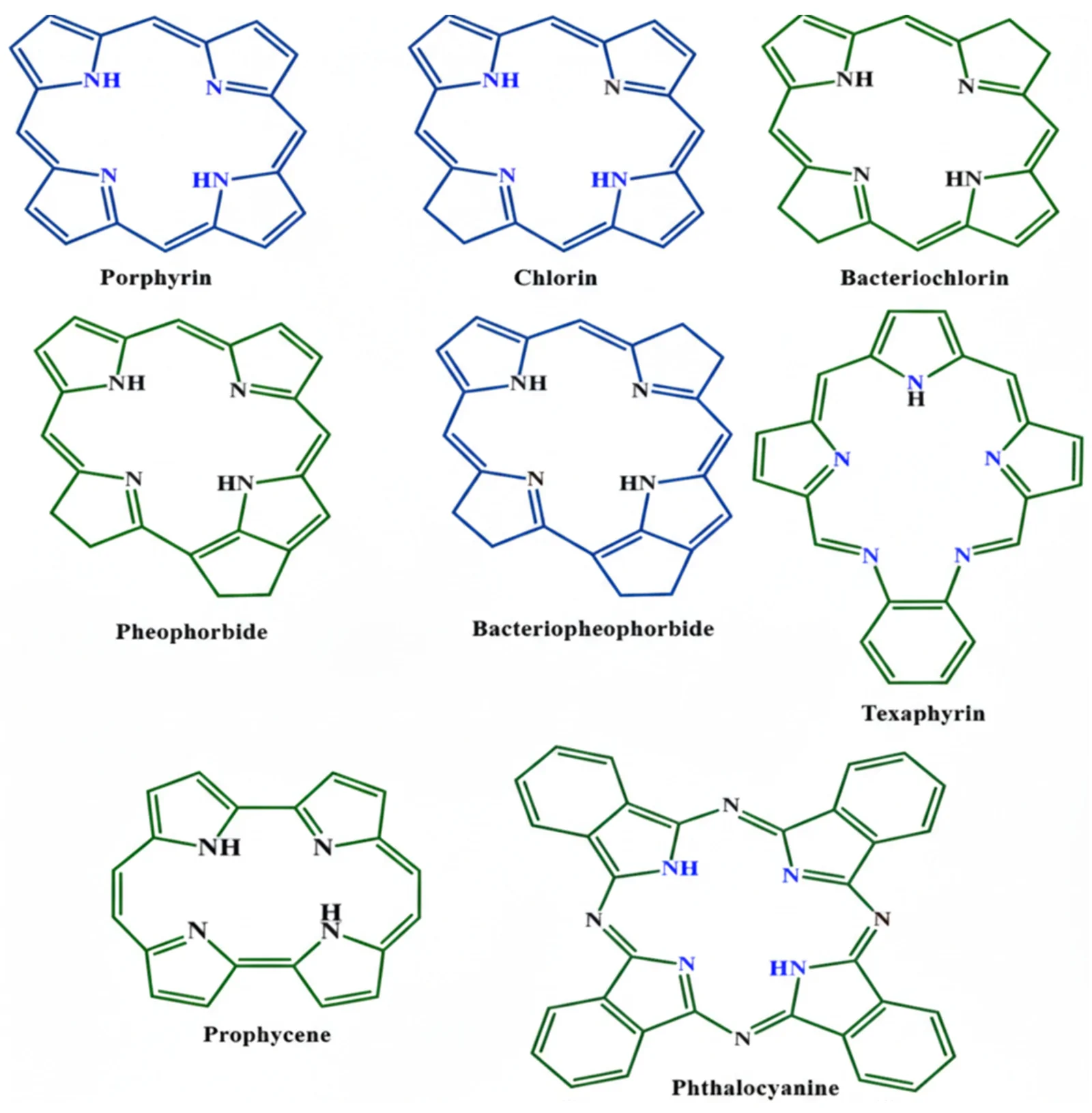
Chemical structure of chlorophyll derivatives.

All natural chlorophyll derivatives are substituted tetrapyrroles with a centrally attached magnesium atom and can be converted into several forms such as chlorins, pheophorbides, bacteriochlorins, porphyrins, bacteriopheophorbides, porphyrins, tex-phyrins, and phthalocyanines (Figure 2). In recent years, chlorophyll derivatives have been widely used in biomedical applications for their antibacterial, anti-inflammatory, antioxidant, anticancer and antimutagenic properties [76,80,81].

**Figure 3:**
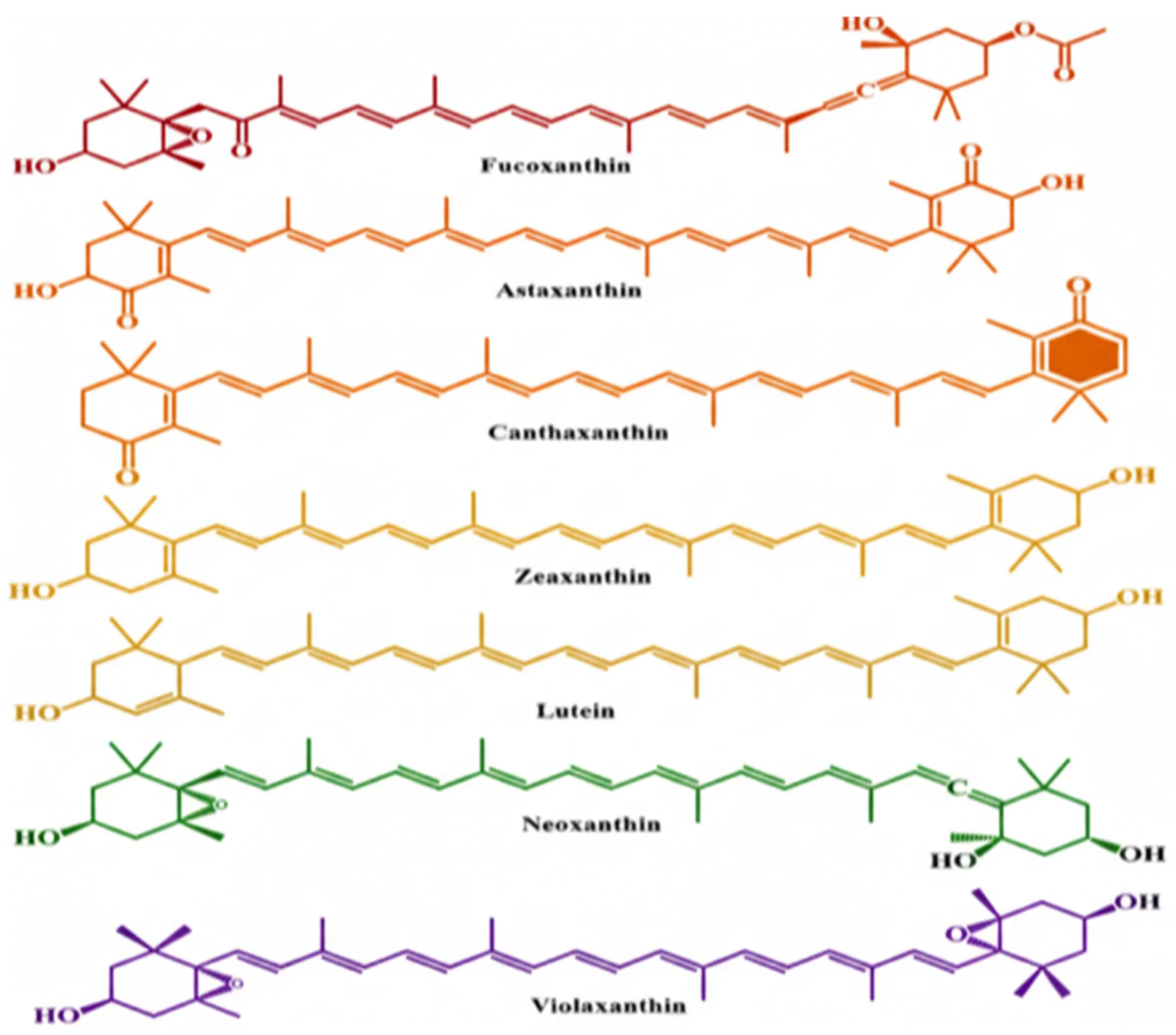
Chemical structure of carotenoids.

Carotenoids are yellow to orange–red pigments that are extensively found in nature and are potential sources of biologically active secondary metabolites. Carotenoids are divided into two groups: carotenes, which are unsaturated hydrocarbons, and xanthophylls, which have one or more oxygen-containing functional groups [82]. Carotenoids are generally used as food colorants and pigments in feeds and have various bioactivities including anticancer, antioxidant and antiviral activities [83,84]. Astaxanthin, fucoxanthin, zeaxanthin, canthaxanthin, neoxanthin, and lutein are carotenoids commonly found in algae (Figure 3). Astaxanthin is an effective pigment in wound healing, and a study found that it showed significant wound closure on the 3rd day of healing and complete wound closure on the 9th day [85]. Another study determined that astaxanthin provided protection against early burn wound progression in a rat skin burn model [86]. Studies have indicated that astaxanthin contributes to diabetic wound healing by inhibiting the expression of inflammatory cytokines such as COX-2, TNF-α, IL-6 and IL-1β [12].

**Figure 4:**
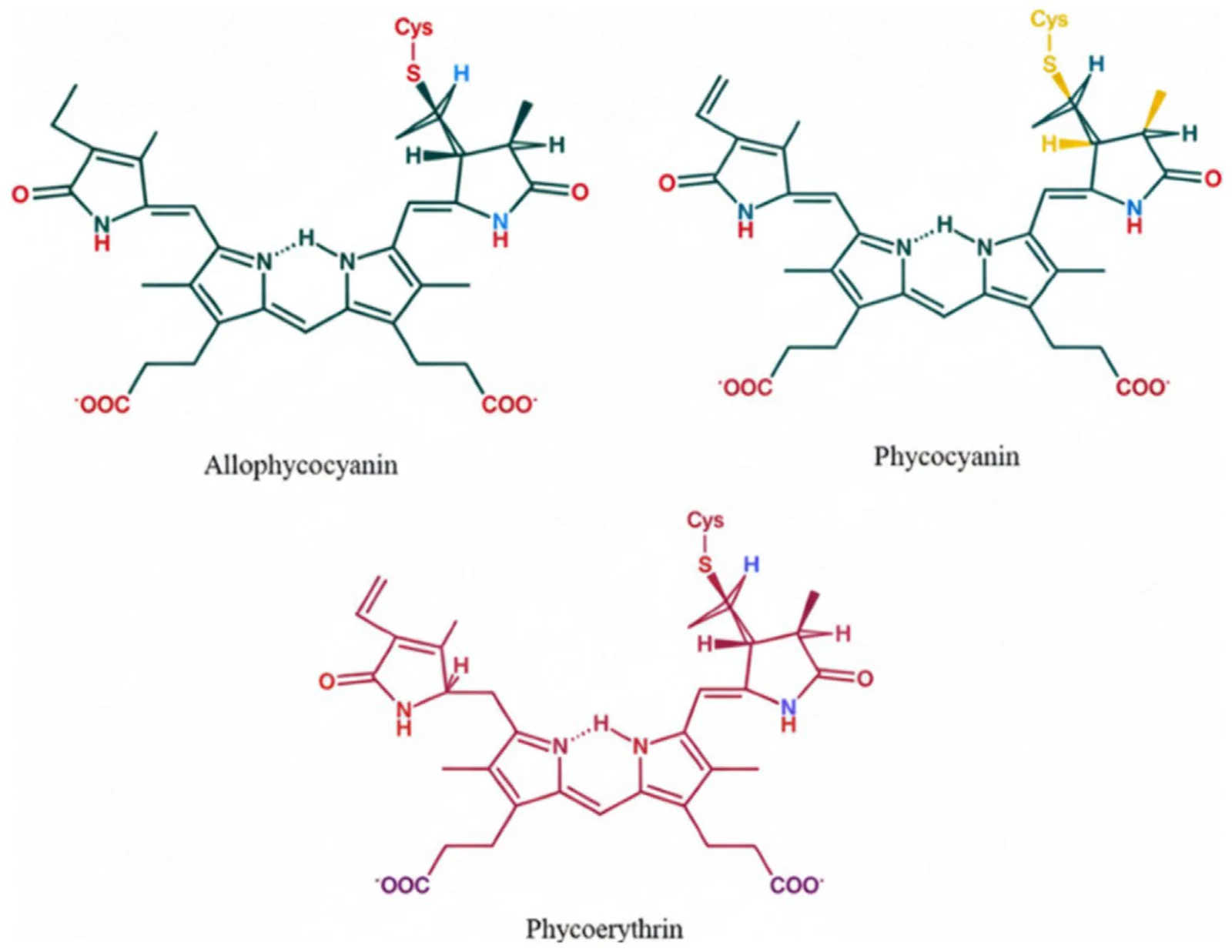
Chemical structure of phycobiliproteins.

Phycobiliprotein is divided into three classes, namely phycoerythrins, phycocyanins, and allophycocyanins, as shown in Figure 4. Phycoerythrins are bright pink, phycocyanins are dark blue, and allophycocyanins are brighter aqua blue pigments [87,88]. C-phycocyanin in *Spirulina* sp. extract was investigated in human keratinocytes using wound in vitro and in vivo models and its positive effects on growth factors in wound healing were reported [89]. Phycobilins have been reported to increase angiogenesis and collagen synthesis in wound healing [12].

**Table 3:** Algal pigments and their effects on wound healing.

Pigments	Algae	Effect on Wound Healing	References
Astaxanthin	*Haematococcus pluvialis*, *Chlorella* spp., *Chlorococcus* spp.	Inhibits the expression of inflammatory cytokines (COX-2, TNF-α, IL-6 and IL-1β)	[90]
β-Carotene	*Dunaliella salina*, *Scenedesmus almeriensis*, *Dunaliella bardawil*, *Chlorella vulgaris*	Increases tissue integrity and maintains cell activity	[91]
Phycobilin	*Spirulina* sp.	Increases collagen synthesis and angiogenesis	[92]
Chlorophyll *a*	All algae	Increases the production of anti-inflammatory cytokines	[93]
Phycocyanin	*Spirulina platensis,* *Arthrospira platensis*	Increases fibroblast proliferation and induces cellular migration to heal the injured area	[94]
Allophycocyanin	*Nostoc* sp.	Provides oxygen delivery to the wound area and encourages cell growth	[12]

### 4.4. Algal Polysaccharides

Bioactive compounds such as agaroid, alginate, fucoidan, carrageenan, laminarins and ulvan polysaccharides have potential in algal wound healing. These polysaccharides are important compounds that are abundant in algae, environmentally friendly and low in cost. Algal polysaccharides are characterized by treatment safety and therapeutic efficacy in wound healing by reducing the negative effects of protease activity and stimulating oxygen permeability and cytokine production. Alginate, fucoidan and laminarin are usually found in brown algae. Agar and carrageenan are more commonly found in red algae, and ulvan is usually found in green algae [95]. Polysaccharide bioactive compounds contained in some algae and their various effective roles in wound healing are listed in Table 4.

### 4.5. Alginate

**Figure 5:**
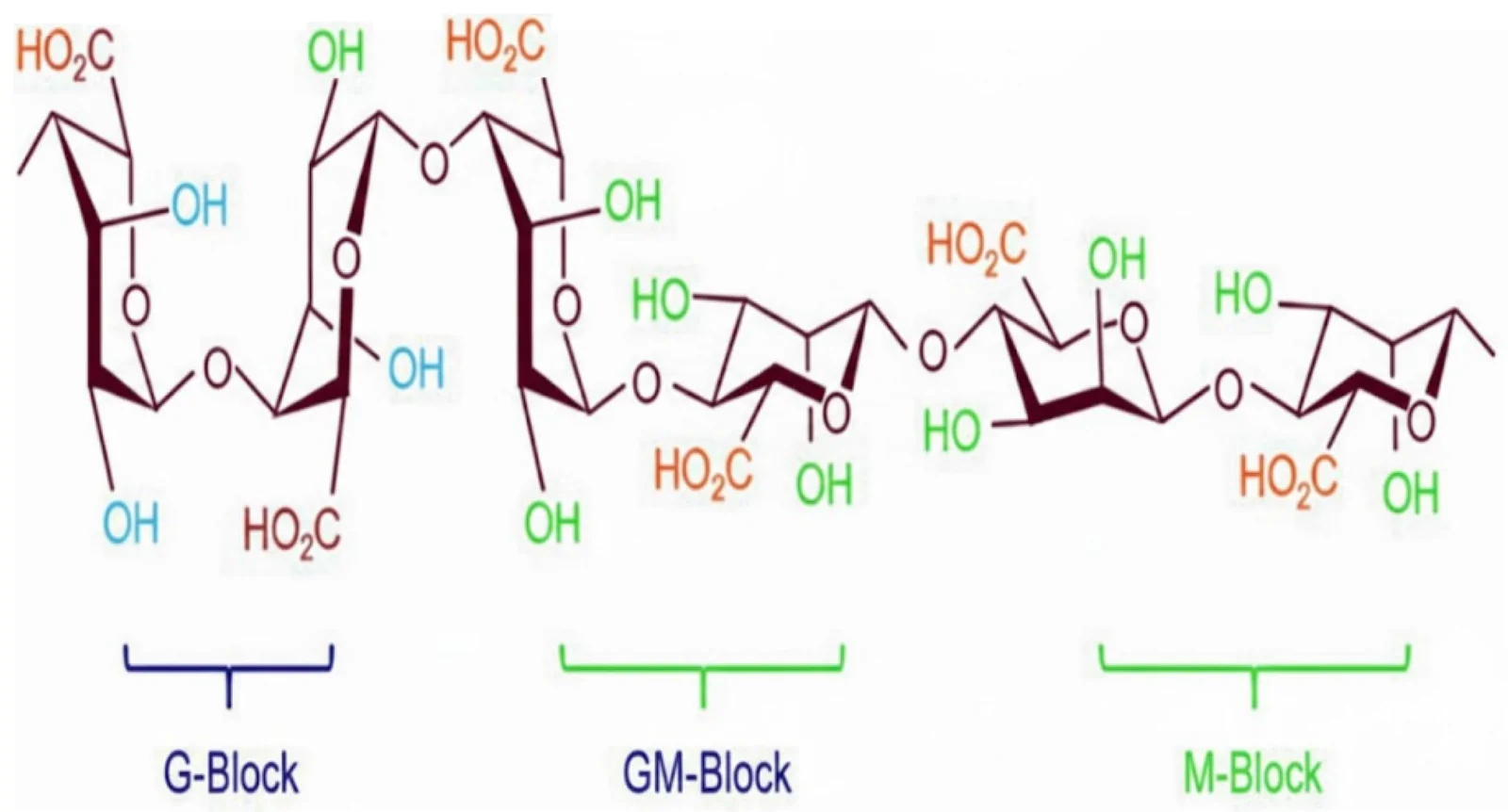
Chemical structure of alginic acid.

Alginate is a polysaccharide composed of β-D-mannuronic acid (M) and its C5 epimer α-L-guluronic acid (G) is obtained from the cell wall of brown algae (*Macrocystis pyrifera*, *Laminaria digitata*, *Laminaria japonica*, *Laminaria hyperborea*). It is known to be a family of copolymers by connecting long sequences of G blocks, M blocks and MG blocks in the polymer chain. Figure 5 shows the chemical structures of β-D-mannuronic acid (M) and α-L-guluronic acid (G) [97,98].

Alginates have been beneficial to the food industry for many years, but in recent years, their use and importance in biomedical applications, medicine and pharmaceutical fields has increased. With their hydrophilic and porous structures, proteins are released quickly from alginate. It provides a moist healing environment on the skin and facilitates healing by reducing infection in the wound area. Alginate, commonly used as a wound dressing, can be applied to the wound site in various forms (foam, hydrogel, film, sponge, nanofiber, membrane), including through anti-inflammatory drugs, antibiotics, and nanoparticles. It can be gelled with calcium, barium, magnesium, nickel, cobalt, zinc, cadmium, lead, strontium, and manganese ions and can promote re-epithelialization and granulation tissue formation in rapid wound healing [99]. Alginate-based wound dressings have several valuable properties compared to traditional wound dressings such as gauze in preventing pathogens from entering the wound and facilitating wound healing. The immunogenic effect of alginate is due to the amount of M block. Alginate with high M block induces cytokine production compared to high G block [100]. Alginate dressings and scaffolds have been shown to reduce wound closure time, decrease the expression of fibrogenic factors and increase collagen type I expression in rat skin wounds [101]. It has also been reported that alginate treatment supports re-epithelialization by increasing collagen-I expression in a rat model of diabetic wound healing [96].

### 4.6. Agaroid (Agar/Agarose)

**Figure 6:**
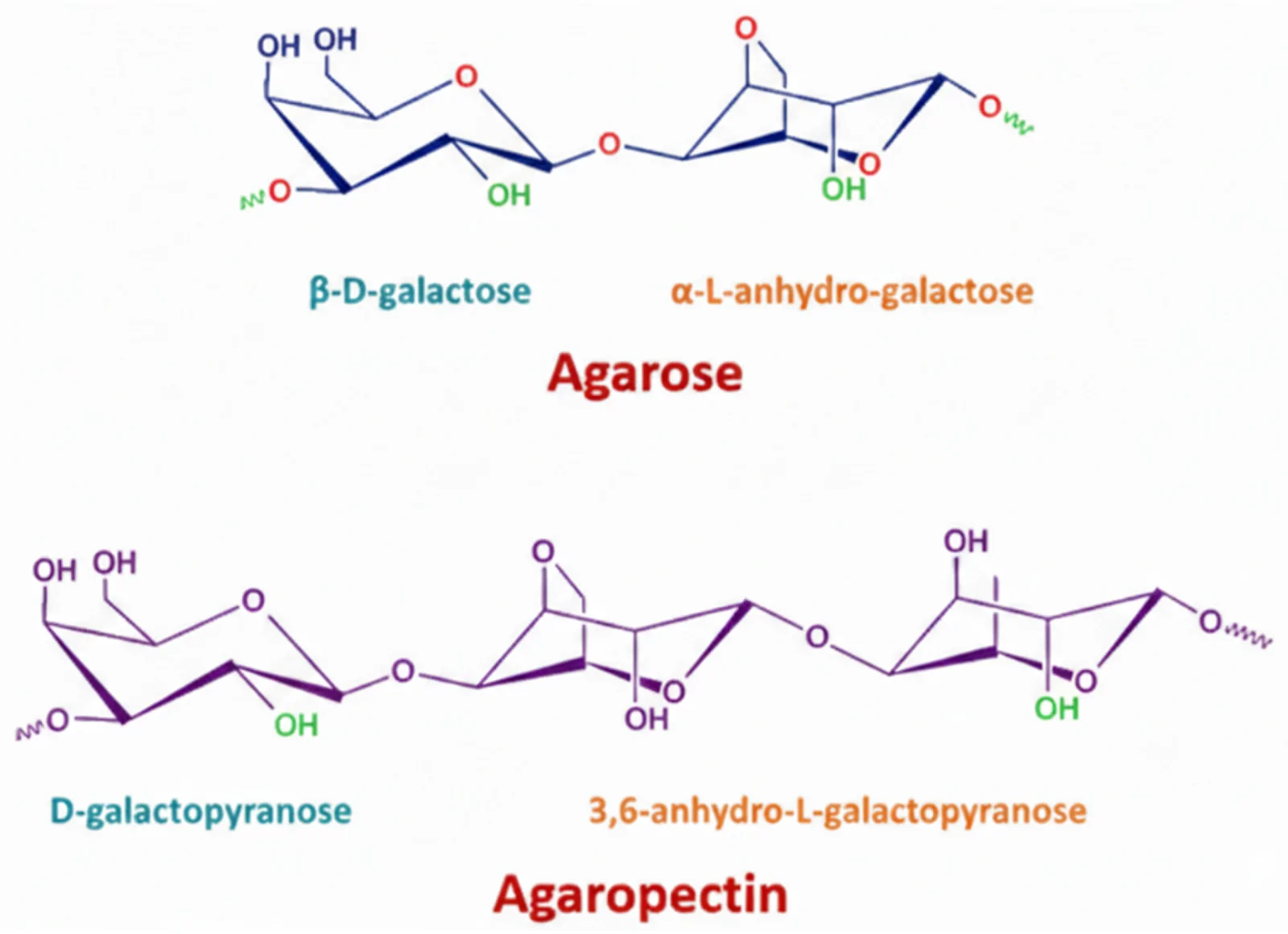
Chemical structure of agar.

Agar, a highly hydrophilic polymer with current functions in food and electrophoresis analysis, has a specific gel property and promising biocompatibility. Agar is composed of a mixture of two polysaccharides: agarose and agaropectin [102]. Agarose is a linear polysaccharide composed of repeating agarobiose units, which consist of β-D-galactose and α-L-anhydro-galactose linked alternately by β-(1→4) and α-(1→3) glycosidic bonds. Agaropectin shares this fundamental backbone of D-galactopyranose and 3,6-anhydro-L-galactopyranose units as shown in Figure 6 [103,104].

Agar obtained from red algae such as *Gracilaria lemaneiformis*, *Porphyra haitanensis*, and *Gelidium amansii* is used in skin dressings. Slow to degrade and lacking cell recognition motifs, agaroids are used as biomaterials by chemical cross-linking. They can be formed in various admixtures with different biopolymers and consist of collagen and gelatin to promote cellular adhesion for wound dressing. In a study, a skin wound dressing was developed by mixing 1–2% agar and 1% collagen type I in different proportions [105]. Another study tested agar and collagen cross-linked composites as wound dressings on rabbit skin lesions, concluding that they supported suitable tissue repair with minimal scarring, fluid exudation, and no illness. In a study of skin regeneration, a hydrogel made of chitosan and agarose (3% *w*/*v*) was produced and evaluated in full-thickness skin wounds in rats. As a result of the study, it was suggested that the group treated with chitosan–agarose hydrogel had no inflammatory reaction and showed better healing, making it suitable as a wound dressing [106].

Although agarose forms a stable, highly porous, three-dimensional (3D) hydrogel network capable of absorbing large volumes of wound exudate and maintaining a moist healing microenvironment, its clinical utility as a standalone matrix is limited due to its biologically inert nature and lack of cell recognition motifs (such as RGD sequences). To overcome this structural drawback, agarose is frequently blended with bioactive natural polymers—such as chitosan or collagen—to create functional composite hydrogel structures. In these composite systems, chitosan plays a complementary biochemical role; under physiological conditions, its protonated primary amino groups impart a positive surface charge that facilitates electrostatic attraction to negatively charged cell membranes and extracellular matrix proteins (e.g., fibronectin and vitronectin). This charge-mediated interaction directly stimulates focal adhesion formation, cell attachment, and the subsequent processes of proliferation and migration in dermal fibroblasts and keratinocytes. Simultaneously, the agarose component preserves the hydrogel’s structural integrity, high water-retention capacity, and controlled swelling kinetics. Consequently, agarose–chitosan composite hydrogel systems successfully combine the mechanical robustness and moisture-regulating capabilities of agarose with the cell-instructive and antimicrobial properties of chitosan, thereby providing an ideal microenvironment for rapid tissue regeneration [107,108].

### 4.7. Carrageenans

**Figure 7:**
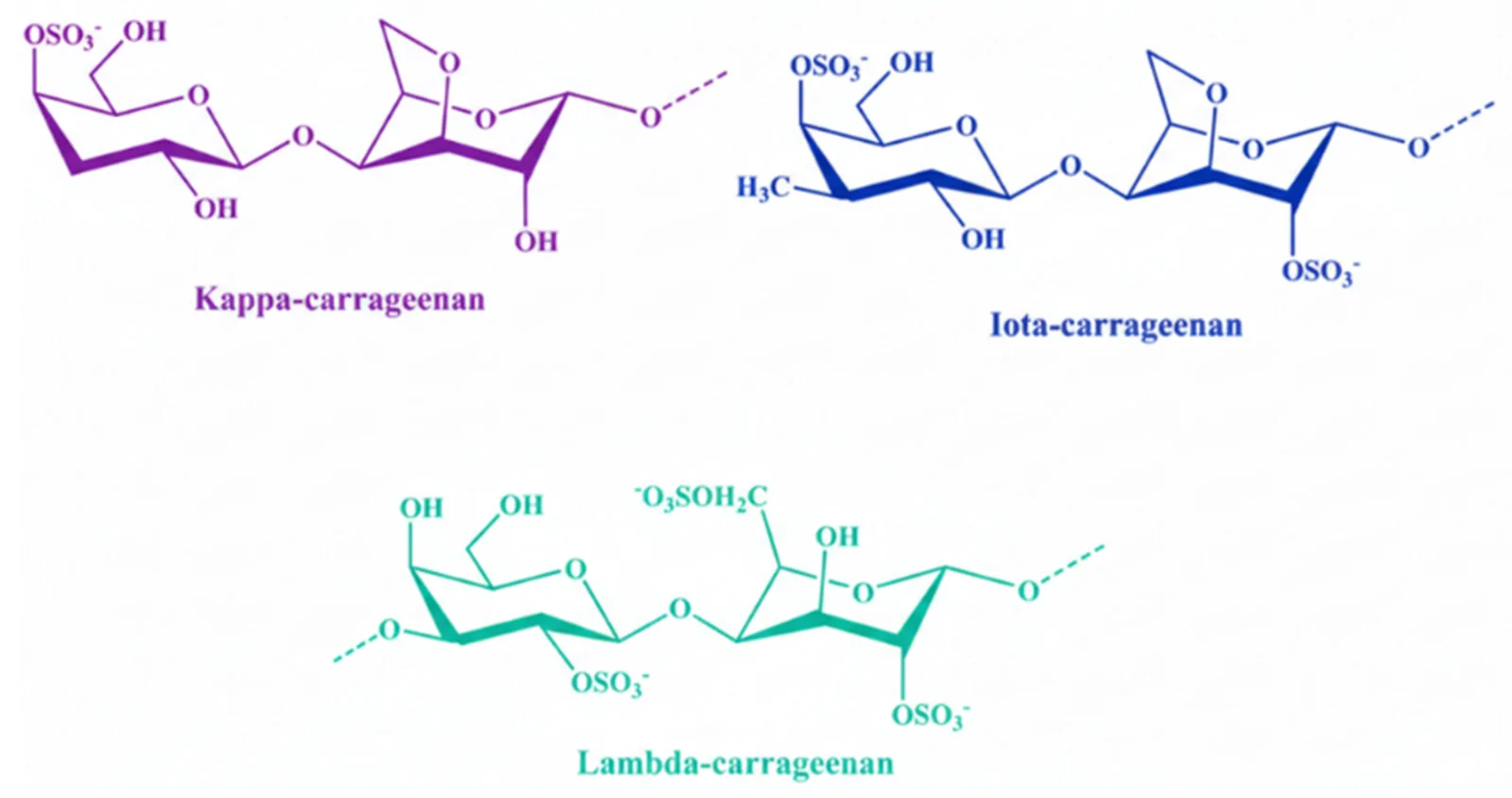
Chemical structural fragments of carrageenans.

The chemical structure of carrageenans, which are mostly used in the food industry, is a sulfated polysaccharide based on a repeating disaccharide unit composed of D-galactose residues linked by β-1→4- and -1→3-glycosidic bonds. They exist in various types depending on the number and position of sulfate groups and the presence of 3–6 anhydro-galactose bridges called kappa (κ), iota (ι) and lambda (λ) (Figure 7). They are water-soluble polysaccharides due to their high hydroxyl and sulfate groups [97,109]. These chemical variations directly dictate their mechanical behavior, blood coagulation response, and anti-inflammatory efficacy in wound management. λ-carrageenan exhibits strong heparin-like anticoagulant properties by inactivating thrombin, whereas κ-carrageenan (1 sulfate group) promotes rapid platelet aggregation and clot formation. Higher sulfation (λ > ι > κ) correlates directly with stronger ROS-scavenging capabilities and down-regulation of proinflammatory cytokines. κ-carrageenan forms rigid, brittle hydrogels due to its 3,6-anhydro-D-galactose ring enabling double-helix junction zones, and ι-carrageenan yields elastic, clear gels, while non-gelling λ-carrageenan is primarily utilized as a viscosity enhancer or bioactive additive [110].

Carrageenan has been obtained from the red algae *Betaphycus gelatinum*, *Chondrus crispus*, *Eucheuma denticulatum*, *Gigartine skottsbergii*, *Kappaphycus alvarezii*, *Hypnea musciformis*, *Mastocarpus stellatus*, *Mazzaella laminaroides*, *Sarcothalia crispate* and *Sarcopeltis skottsbergii* [111]. Carrageenans have been found to have various biological functions such as antibacterial, antiviral, anti-inflammatory and immunomodulatory [112]. One study presented that carrageenan and xylan mixed in different proportions had high anti-inflammatory and wound healing potential [113].

The physicochemical properties of carrageenans permit them to be merged with other materials to form hydrogel systems. Gel properties depend on the position and amount of sulfate esters [114]. There is evidence on the function of carrageenan-based hydrogels for wound healing dressing materials. In recent studies, dressings designed with kappa (K) carrageenan and sodium alginate with excellent functional properties for chronic wounds are being prepared [115]. In one study, poly(N-vinyl-2-pyrrolidone), potassium chloride and K-carrageenan hydrogels used in wound healing showed significant results such as biocompatibility, prevention of bacterial infections, flexibility, and elasticity [116].

### 4.8. Fucoidans

**Figure 8:**
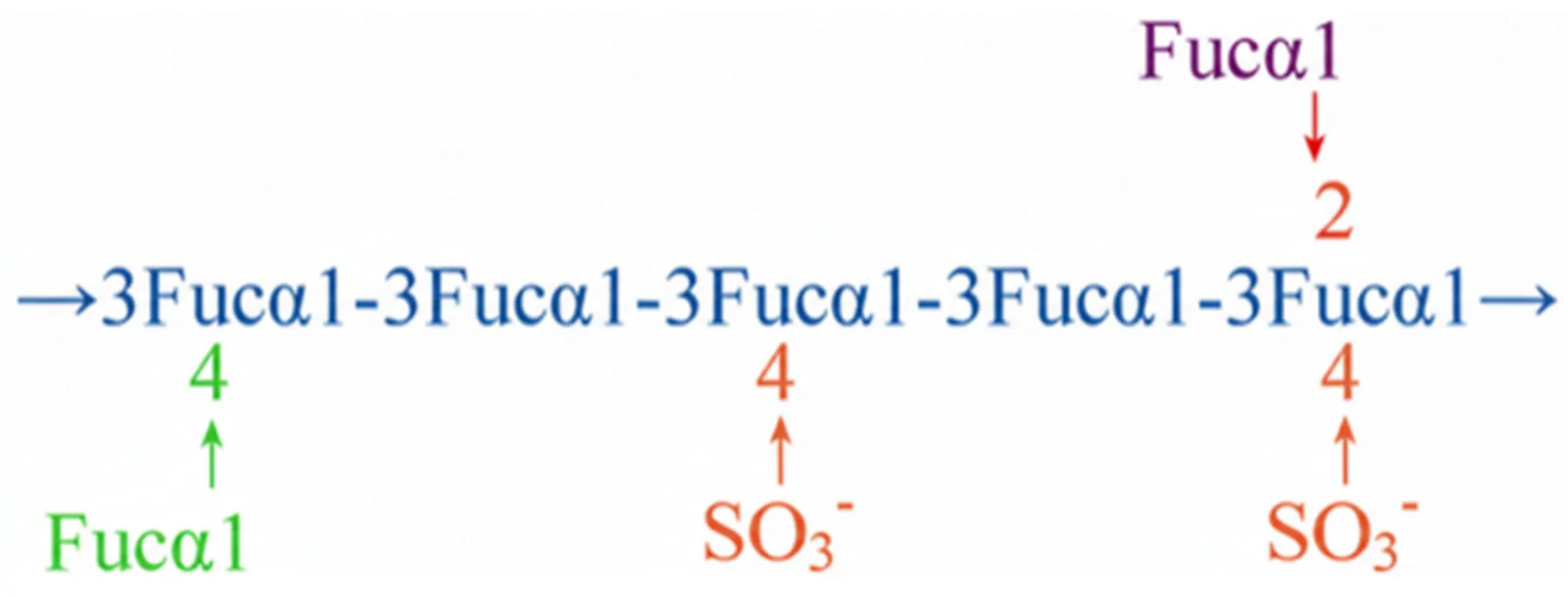
Chemical structure of fucoidan according to the Patankar model.

Fucoidans are polymers derived from brown sea algae in which fucose forms the core monomeric module. Patankar proposed that the core region of fucoidan is primarily an α-(1→3)-linked fucose polymer substituted with sulfate groups at the C-4 position of some fucose residues [117,118,119,120]. Fucose also binds to this polymer, creating a branch point for every 2–3 fucose residues in the chain (Figure 8).

The chemical structure of fucoidan in different brown algae is complex, while fucoidan in *Fucus vesiculosus* has a simple chemical structure consisting of fucose and sulfate [121]. Apart from fucose and sulphate, they may contain monosaccharides such as glucose, galactose, xylose, mannose, uronic acids, and acetyl groups [119]. The structures of fucoidans from different brown algae vary depending on the species, and Table 5 shows the fucoidan structures in various brown algae.

Fucoidans isolated from different species have various biological activities such as antioxidant, antibacterial, anticoagulant, antitumor, antivirus, anti-inflammatory and antithrombotic [112]. In addition, fucoidan regulates various growth factors on wound healing and is beneficial in wound treatment. For example, fucoidans have been reported to increase fibroblast growth factor (FGF) activity and modulate the effects of TGF-β1 on wound healing [122,123]. In one study, fucoidan–chitosan hydrogel formulations were found to be good for the treatment of rabbit dermal burns by promoting re-epithelialization of the wound area [124]. Another study concluded that sponge containing alginate/chitosan/fucoidan (ACF, 10% fucoidan) was effective against *Staphylococcus aureus* and *Escherichia coli* and notably accelerated wound closure in a rat model. It has also been found that ACF helps with collagen production in the dermis, hair follicle regeneration and reducing inflammation [125]. Studies have reported that fucoidan reduces lipid peroxidation and strengthens the antioxidant defense system [126].

### 4.9. Ulvans

**Figure 9:**
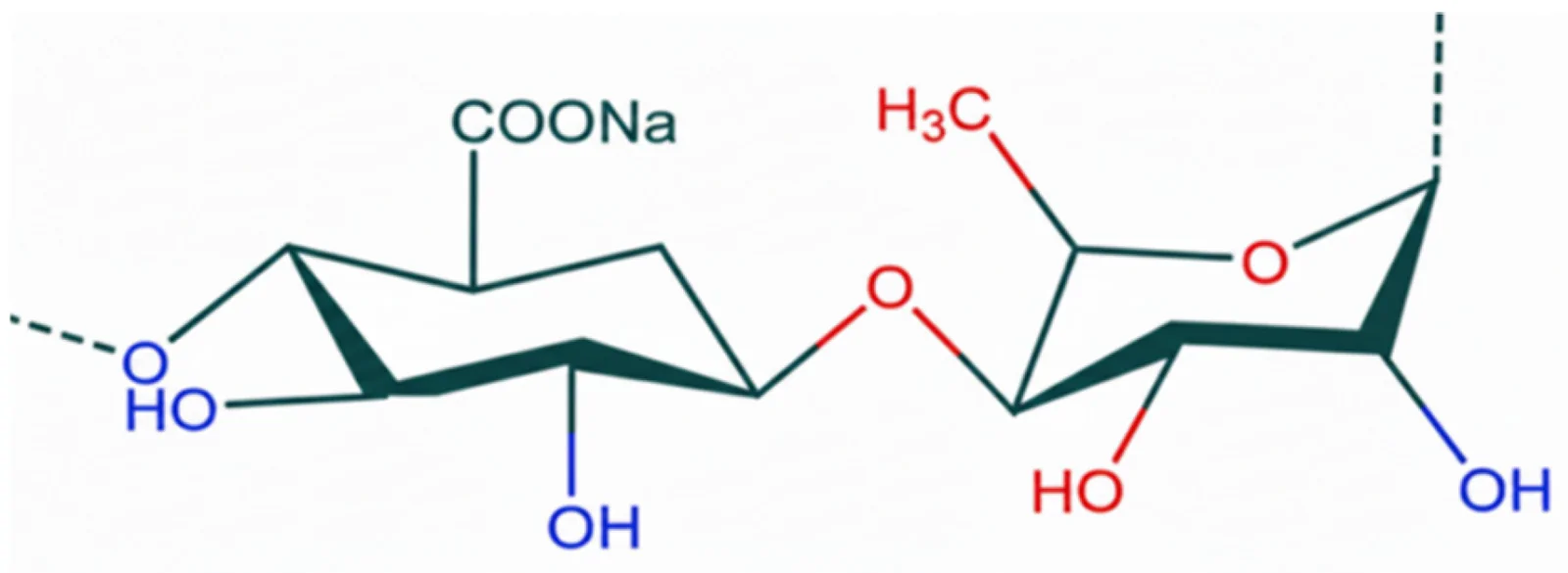
Chemical structure of ulvans.

Ulvans are anionic sulfated heteropolysaccharides isolated from green algae such as *Ulva australis*, *U. compressa*, *U. ralfsii*, *U. prolifera*, *U. rigida*, *U. flexuosa*, and comprising rhamnose, uronic acid and xylose as the chief monomeric sugars (Figure 9). Ulvans also serve as a potential source of iduronic acid used in the synthesis of heparin fragment analogs with antithrombotic activities [127,128].

Table 6 shows the chemical structure contents of ulvans consisting mainly of rhamnose in *Ulva* sp. It differs from other polysaccharides due to the complexity of glycosidic bonds and group modifications of the ulvans, the main sugar molecules of which are xylose, highly sulfated rhamnose, iduronic and glucuronic acid [97,129].

Ulvans, which share key functional features with glycosaminoglycans and heparins despite having distinct rhamnose-based backbones, are effectively used in biomedical applications. Ulvans inhibit thrombin formation by inducing fibroblast proliferation and are frequently used in the healing process by improving fibrinolytic function. In addition, they stimulate cell proliferation and collagen biosynthesis because they are polysaccharides rich in rhamnose compounds. In recent years, the antiviral, antioxidant, anti-inflammatory and anticoagulant activities of ulvan have been reported by researchers [131]. It has also been reported that ulvan has protective activities against bacterial attacks that may occur in wound conditions. Ulvan, mentioned as an anti-inflammatory agent, significantly increases the secretion of proinflammatory cytokines, namely interleukin-1β (IL-1β), interleukin-6 (IL-6), interleukin-12 (IL-12) and tumor necrotizing factor-alpha (TNF-α) and supports angiogenesis by triggering fibroblasts for collagen production [132]. Additionally, ulvan-based membranes have been produced for wound dressings as carriers of therapeutic agents. A new study concluded that ulvan-based nanofibrous patches enhanced wound healing in skin trauma resulting from cryosurgical treatment of keloids [133]. In another study, ulvan obtained from *Ulva fenestrata* was used as a hydrogel in chronic diabetic wound healing [96].

### 4.10. Laminarin

**Figure 10:**
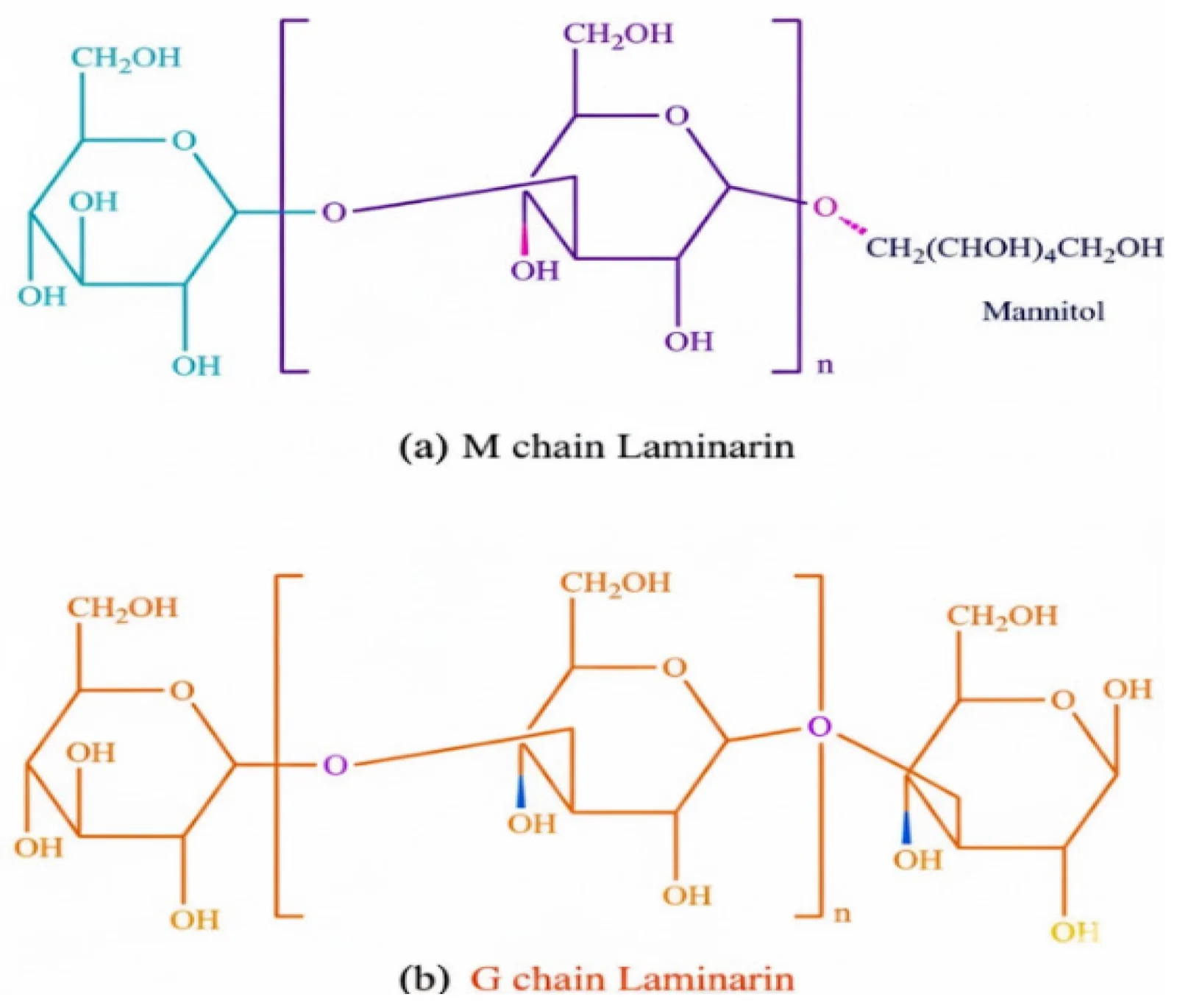
Chemical structure of laminarin.

Laminarin is an important water-soluble polysaccharide consisting of (1,3)-β-D-glucan with β (1,6) branching and containing 20-25 glucose units. Compared to other polysaccharides, the polymer chains of laminarins, which have a lower average molecular weight, also differ at the reducing end. The M-type chain terminates with a 1-O-substituted D-Mannitol group, while the G-type chain terminates with D-glucose units (Figure 10). The chain structure of the two types of laminarin may vary depending on the algae species and environmental factors [128,134,135,136].

Laminarin, which is abundant in brown algae such as *Laminaria* sp., *Saccharina* sp., and *Eisenia* sp., has been reported to exhibit antiapoptotic, antioxidant, anticoagulant, and anti-inflammatory activities. Laminarin has been reported to have antitumor, anticoagulant, antibacterial, immune-boosting, and blood pressure-, cholesterol- and triglyceride-lowering properties [137]. The promising results of laminarin and laminarin-based materials in wound healing are that they stimulate dermal regeneration and cellular proliferation and migration in experiments with mice [136,138]. It has been shown that topical application of laminarin-based creams on wounds provides protection against bacteria and free radical oxidative damage, while accelerating collagen accumulation and re-epithelization, thus shortening the healing process [139].

## 5. Conclusions and Future Perspectives

Algae have gained significant momentum across various sectors, particularly within the food, cosmetic, and pharmaceutical industries. Recently, there has been a notable surge in research dedicated to isolating and characterizing algal bioactive molecules, which offer promising potential for improving human health, preventing disease, and serving as high-value biomedical raw materials. As highlighted throughout this review, algal bioactive metabolites—including polysaccharides, pigments, peptides, and fatty acids—exhibit exceptional therapeutic properties that directly modulate the fundamental phases of cutaneous tissue repair through antimicrobial, antioxidant, anti-inflammatory, and pro-angiogenic mechanisms. Pre-clinical in vitro and in vivo studies consistently validate their capacity to accelerate re-epithelialization, promote fibroblast proliferation, and regulate key signaling pathways such as TGF-β, VEGF, and TNF-α. Consequently, these marine-derived biopolymers serve as crucial structural and functional components in the development of next-generation wound dressings, hydrogels, and electrospun nanofibrous scaffolds. However, despite these encouraging pre-clinical outcomes, transitioning algal biocompounds from laboratory benchtop research to commercial, FDA/EMA-approved clinical applications faces key pharmacological and translational hurdles. To successfully bridge this gap, future perspectives must prioritize addressing the biochemical safety, immunogenicity, manufacturing consistency, and regulatory compliance of raw algal materials. First, marine algae naturally bioaccumulate trace heavy metals (e.g., inorganic arsenic, cadmium, lead) and environmental pollutants from seawater, while crude extracts may contain pyrogenic bacterial endotoxins or cellular residues. Establishing standardized, multi-step industrial purification protocols (such as ultrafiltration and ion-exchange chromatography) is imperative to guarantee that contaminant levels remain strictly below clinical safety limits (<0.5 EU/mL).

Second, while purified sulfated polysaccharides generally exert anti-inflammatory effects, unfractionated high-molecular-weight fractions or structural impurities can inadvertently activate toll-like receptors and induce unwanted immunogenic foreign-body responses; applying controlled enzymatic depolymerization to produce low-molecular-weight derivatives offers a viable solution to eliminate immunogenicity without compromising biological potency. Furthermore, the intrinsic batch-to-batch variability of natural algal biomass—driven by seasonal, geographical, and species-dependent environmental fluctuations—presents a major bottleneck for industrial scale-up. Overcoming this limitation mandates the implementation of validated analytical quality-control benchmarks, including High-Performance Anion-Exchange Chromatography (HPAEC), Nuclear Magnetic Resonance (NMR) spectroscopy, and Gel Permeation Chromatography (GPC), to ensure rigorous batch-to-batch chemical reproducibility.

Finally, future studies must evaluate the compatibility of terminal sterilization techniques (e.g., gamma irradiation vs. ethylene oxide) to ensure they do not alter or degrade delicate biopolymer matrices, while strictly adhering to ISO 10993 standards [140] for biological safety evaluation. By systematically resolving these pharmacological, toxicological, and regulatory challenges, the research highlighted in this review will lay a solid foundation for future scientific breakthroughs and accelerate the commercial translation of algal bioactive metabolites into advanced, clinically effective wound care technologies.

## Figures and Tables

**Table 1:** Algae containing pharmacological secondary metabolites.

Metabolites	Algae	Pharmacological Activities	References
Apratoxin E	*Lyngbya bouillonii*	Cytotoxicity	[31]
Itralamide B	*Lyngbya majuscula*	Cytotoxicity	[32]
Tuberatolide B	*Sargassum macrocarpum*	Anticancer	[33]
Aurilide	*Lyngbya sordida*	Anticancer	[34]
Laucysteinamide A	*Caldora penicillata*	Antitubilin	[35]
Dolastatin	*Caldora penicillata*	Anticancer and cytotoxic	[36]
Dieckol	*Ecklonia stolonifera*	Anticancer	[37]
Noscomin	*Nostoc commune*	Antibacterial	[38]
Muscoride A	*Nostoc muscorum*	Antibacterial	[39]
Nostoflan	*Nostoc flagelliforme*	Antiviral	[40]
Tolybyssidins	*Tolypothrix byssoidea*	Antifungal	[41]
Schizotrin A	*Schizothrix* sp.	Antifungal	[42]
Cyanovirin-N	*Nostoc ellipsosporum*	Anti-HIV	[43,44]
Scytovirin	*Scytonema varium*	Anti-HIV	[45]
Stigmasterol	*Navicula incerta*	Cytotoxicity, apoptosis induction	[46]
Zeaxanthin	*Porphyridium purpureum*	Antiproliferative, apoptosis induction	[47]
Fucoidan	*Turbinaria conoides*	Antiproliferative, apoptosis induction, antioxidant	[48]
Ascophyllan	*Ascophyllum nodosum*	Anti-metastatic	[49]
Chrysolaminarin	*Odontella aurita*	Antioxidant	[50]
Fucoxanthin	*Phaeodactylum tricornutum*	Antioxidant	[51]
Scytonemin	*Leptolyngbya mycodia*	Antioxidant	[52]
Phlorotannins	*Sargassum vulgare*	Antioxidant	[53]
Proanthocyanidins	*Spirogyra varians*	Antimicrobial	[54]
Sulfated galactans	*Botryocladia occidentalis*	Immunostimulatory, antiviral and anticoagulant	[55]
Phycarine	*Laminaria digitata*	Antitumor	[56]
Loliolide	*Undaria pinnatifida*	Antioxidant, bactericidal, and antitumor	[57]
Allophycocyanin	*Spirulina platensis*	Antiviral	[58,59]
Deschloroelatol	*Laurencia rigida*	Antifungal	[60]
Calothrixin-A	*Calothrix* sp.	Antimalarial and anticancer, antiviral	[43]

**Table 4:** Algal polysaccharides effective in wound healing [11,96].

Algae	Species	Bioactive Compounds	Role in Wound Healing
Phaeophyta (Brown algae)	*Laminaria digitata* *Laminaria japonica* *Turbinaria conoides* *Fucus vesiculosus* *Cladosiphon okamuranus* *Undaria pinnatifida*	Fucoidan	Antioxidant, antibacterial, and provides cell proliferation. It regulates various growth factors on wound healing and is beneficial for wound treatment.
Rhodophyta (Red algae)	*Chondrus crispus* *Eucheuma denticulatum* *Gigartine skottsbergii* *Kappaphycus alvarezii* *Hypnea musciformis* *Mastocarpus stellatus* *Mazzaella laminaroides* *Sarcothalia crispata* *Sarcopeltis skottsbergii*	Carrageenan	Antioxidant, antibacterial and anti-inflammatory.
Rhodophyta (Red algae)	*Gracilaria cornea* *Gracilaria dominguensis* *Gracilaria lemaneiformis* *Porphyra haitanensis* *Gelidium amansii*	Agar	It regulates the inflammatory response, is an antioxidant, and facilitates tissue healing.
Phaeophyta (Brown algae)	*Macrocystis pyrifera* *Laminaria digitata* *Laminaria japonica* *Laminaria hyperborea*	Alginate	It has anti-inflammatory, anti-infection, and moisturizing properties and facilitates wound healing.
Phaeophyta (Brown algae)	*Laminaria hyperborea* *Saccharina* sp. *Eisenia* sp.	Laminarin	Antibacterial, antioxidative, and accelerates collagen accumulation and re-epithelization, shortening the healing process.
Chlorophyta (Green algae)	*Ulva lactuca* *Ulva fenestrata*	Ulvan	Antioxidative and accelerates wound healing. Increases fibroblast proliferation. Promotes angiogenesis by significantly increasing the release of proinflammatory cytokines and triggering fibroblasts to produce collagen.

**Table 5:** Chemical compositions of some fucoidans [119].

Brown Algae	Chemical Composition
*Fucus vesiculosus*	fucose, sulfate
*F. evanescens*	fucose, sulfate, acetate
*F. distichus*	fucose, sulfate, acetate
*F. serratus*	fucose, sulfate, acetate
*Lessonia vadosa*	fucose, sulfate
*Macrocystis pyrifera*	fucose, galactose, sulfate
*Pelvetia wrightii*	fucose, galactose, sulfate
*Undaria pinnatifida*	fucose, galactose, sulfate
*Ascophyllum nodosum*	fucose, xylose, glucuronic acid, sulfate
*Himanthalia lorea*	fucose, xylose, glucuronic acid, sulfate
*Bifurcaria bifurcate*	fucose, xylose, glucuronic acid, sulfate
*Padina pavonia*	fucose, xylose, mannose, glucose, galactose, sulfate
*Laminaria angustata*	fucose, galactose, sulfate
*Ecklonia kurome*	fucose, galactose, mannose, xylose, glucuronic acid, sulfate
*Sargassum stenophyllum*	fucose, galactose, mannose, glucuronic acid, glucose, xylose, sulfate
*Adenocytis utricularis*	fucose, galactose, mannose, sulfate
*Hizikia fusiforme*	fucose, galactose, mannose, xylose, glucuronic acid, sulfate
*Dictyota menstrualis*	fucose, xylose, uronic acid, galactose, sulfate
*Spatoglossum schroederi*	fucose, xylose, galactose, sulfate

**Table 6:** Chemical composition of ulvans in different *Ulva* sp. [130].

*Ulva* sp.	Monosaccharide (mol%)
*U. australis*	Rhamnose (51) Glucuronic acid (18) Xylose (22) Iduronic acid (7)
*U. rigida*	Rhamnose (49) Glucuronic acid (26) Xylose (6) Iduronic acid (18)
*U. flexuosa*	Rhamnose (56) Glucuronic acid (21) Xylose (15) Iduronic acid (6)
*U. compressa*	Rhamnose (47) Glucuronic acid (24) Xylose (17) Iduronic acid (7)
*U. prolifera*	Rhamnose (60) Glucuronic acid (17) Xylose (15) Iduronic acid (7)
*U. ralfsii*	Rhamnose (43) Glucuronic acid (26) Xylose (14) Iduronic acid (6) Galactose (10)

## Data Availability

No new data were created or analyzed in this study. Data sharing is not applicable to this article.
